# Instagram's impact on dental consumers: analyzing toothpaste hashtags

**DOI:** 10.3389/froh.2024.1420500

**Published:** 2025-01-07

**Authors:** Khalifa S. Al-Khalifa, Rasha AlSheikh, Basmah O. Alakloby, Hind M. Alharbi, Razan F. Alghamdi, Saqib Ali, Laila Al Dehailan

**Affiliations:** ^1^ Department of Preventive Dental Sciences, College of Dentistry, Imam Abdulrahman Bin Faisal University, Dammam, Saudi Arabia; ^2^ Department of Restorative Dental Sciences, College of Dentistry, Imam Abdulrahman Bin Faisal University, Dammam, Saudi Arabia; ^3^ College of Dentistry, Imam Abdulrahman Bin Faisal University, Dammam, Saudi Arabia; ^4^ Department of Biomedical Dental Sciences, College of Dentistry, Imam Abdulrahman Bin Faisal University, Dammam, Saudi Arabia

**Keywords:** toothpaste, instagram, fluoride, influencers, social media

## Abstract

**Background:**

Toothbrushing is the basic step in maintaining oral hygiene and managing caries. The type of toothpaste used, combined with effective toothbrushing techniques, significantly influences oral health outcomes. Information shared on social media platforms can create awareness, generate interest, and influence perceptions regarding toothpaste brands and their benefits. This raised the necessity to comprehensively understand the influence of social media, particularly Instagram, on consumer decision-making processes and behavior related to toothpaste selection.

**Aim:**

to explore toothpaste-related content on Instagram by addressing this gap and highlighting the characteristics of the top-performing toothpaste posts. Method: data were acquired for the “Top 12 posts” for each selected hashtag listed by the Instagram search algorithm. The contents of each post, including the number of likes, number of followers, content type, poster role, post content, post theme, post type, and account type were collected. Moreover, whether the poster was a dentist, patient, or dental interest group was identified. Data was then analyzed using SPSS with a statistical significance level set at *p* = .05.

**Results:**

The study analyzed a total of 1,054,985 posts revealing varying levels of engagement and content characteristics. Notably, the #Toothpaste hashtag garnered the highest number of posts, while #ToothpasteNatural had the lowest. Posts were predominantly promotional (61.1%) compared to educational (38.9%), with marketing being the primary theme. Educational content attracted significantly more engagement, with more likes/views and comments than promotional content. Additionally, the role of the poster influenced content type, with patients and dentists associated more with educational content, while dental interest groups and companies favored promotional material. However, there was no significant difference in the accuracy of claims between educational and promotional content.

**Conclusion:**

Instagram shapes consumer behavior in toothpaste selection, with promotional content dominating despite higher engagement with educational posts. Limited fact-based content highlights the need for stricter regulations and increased contributions from dental professionals to improve oral health education.

## Introduction

Toothbrushing is the basic step in maintaining oral hygiene and caries management. It eliminates dental biofilm and interrupts the caries initiation/progression process. Toothpaste has been used since ancient times as a cleaning agent; in the recent century, toothpaste has been used as an effective therapeutic agent in maintaining and improving oral health and caries control, especially after the inclusion of fluoride (1). It is worth saying that without the therapeutic effect of fluoridated toothpaste, brushing alone has a very limited effect on caries control (2, 3). Adding therapeutic agents shifted toothpaste from merely cosmetic to essential in maintaining oral health. The type of toothpaste used, combined with effective toothbrushing techniques, significantly influences oral health outcomes (4). Oral health professionals play a crucial role in educating patients on the correct use of fluoride toothpaste to maximize its benefits (5).

Social media platforms have become influential channels that can impact consumer behavior and decision-making processes, including toothpaste selection (6, 7). Adolescents, for example, are influenced by social media when choosing oral hygiene products, including toothpaste (7). The information shared on social media platforms can create awareness, generate interest, and influence perceptions regarding toothpaste brands and their benefits.

The interactive nature of social media allows users to engage with content, share experiences, and seek advice from peers or influencers regarding toothpaste selection. Social media influencers, who have a substantial following and credibility in specific niches, can also impact consumer choices by endorsing or reviewing toothpaste products (8).

Instagram is a powerful tool for educating the public on diverse oral health topics, simplifying complex information, and enhancing public understanding of critical oral health practices (9). Patients need to seek advice from credible resources to make educated decisions regarding their health. In Saudi Arabia, for example, patients showed interest in communicating with their dentists via Instagram, using channels like direct messages or posting comments/stories; this includes seeking advice on purchasing dental products such as toothpaste (10, 11). However, it is crucial to acknowledge the potential limitations of Instagram as a sole source of dental information. The platform's inherent emphasis on visuals and brevity can lead to oversimplification of complex topics and potentially misinformation from unverified sources.

This raised the necessity to comprehensively understand the influence of social media, particularly Instagram, on consumer decision-making processes and behavior related to toothpaste selection. There is a lack of in-depth assessment of the specific characteristics of top-performing (most liked) toothpaste posts on Instagram and how these factors influence consumer choices. This study aimed to explore toothpaste-related content on Instagram by addressing this gap and highlighting the characteristics of the top-performing toothpaste posts (12). The null hypothesis was that no difference would be found between the toothpaste-related Instagram posts regarding the credentials, post format, content, and accuracy of claims.

## Methodology

**Figure 1:**
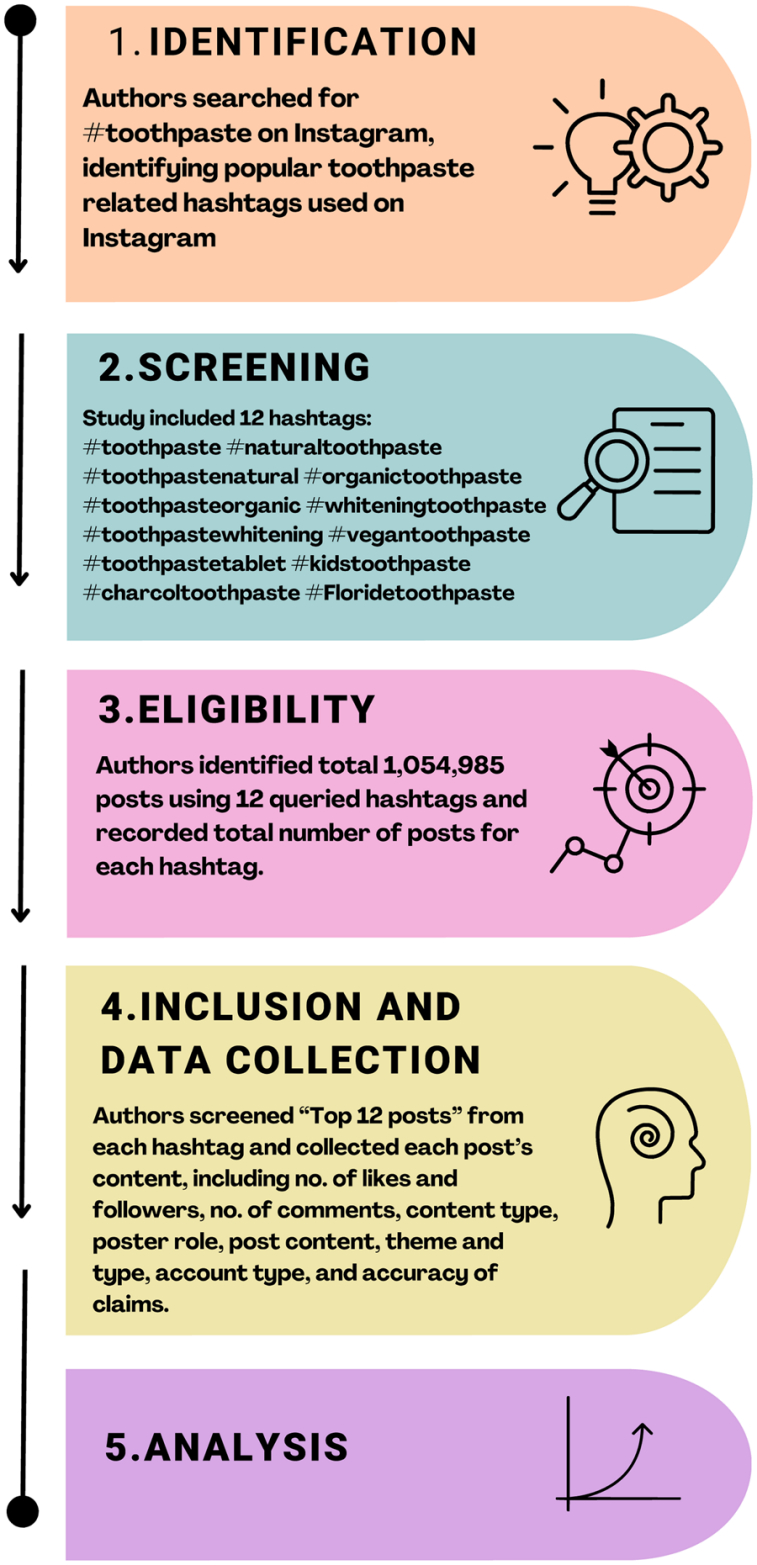
Flow chart of analyzing toothpaste-related content on Instagram.

This observational cross-section study was analyzed publicly with data derived from Instagram. The study design is illustrated in Figure 1. Upon the hashtag selection, we installed certain search methods and data analysis procedures established in prior research (12, 13).

### Data collection

**Figure 2:**
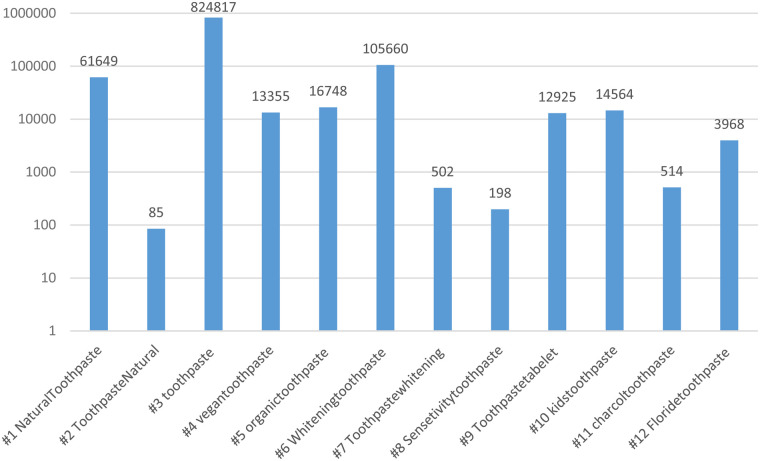
Number of posts per toothpaste-related hashtags on Instagram. A Logarithmic scale graph (Base 10).

Two researchers (B.A. and H.A.) independently reviewed a comprehensive list of hashtags related to toothpaste on Instagram on January 2nd, 2024. The final list of 12 hashtags (presented in Figure 2) was chosen after an initial exploration of broader terms like “#toothpaste” and “#dentalcare”. Both researchers conducted a pilot assessment on a sample of posts to establish shared evaluation criteria. Any disagreements during post-evaluation were resolved through discussion to reach a consensus.

On the same day, the “Top 12 posts” for each selected hashtag were retrieved based on the Instagram search algorithm. Hashtags related to toothpaste were identified through an initial exploration of broad terms like #Toothpaste and #DentalCare. From this, 12 specific hashtags were selected based on their relevance and prevalence, as determined by Instagram's search algorithm. The “Top 12 posts” for each hashtag were chosen to focus on the most visible and engaging content, as these posts are most likely to influence consumer behavior and reflect trends in promotional and educational strategies. This approach ensured the study targeted content with the highest potential impact on audience perceptions. From each post, various data points were collected, including the number of views and likes and the account followers, comments, and content (image or video). Other information collected were the of poster role (patient, dentist, dental organization, or dental company), the idea behind each post content (educational or promotional), the post theme (marketing, informational, or experience sharing), post type (clinical procedure, product advertisement, or practice advertisement), and the account type (dental professional, company, dental lab, dental practice, or influencer). Additionally, we categorized posts as educational if they primarily aimed to educate or raise awareness about toothpaste-related topics. Promotional posts refer to those promoting dental professionals, practices, or advertising specific procedures or products. Finally, one researcher (R.A.) evaluated the accuracy of claims (factual vs. non-factual) regarding the safety of toothpaste or related interventions and their efficiency and effectiveness. A simplified 2-point scale adapted from Alkhadimi et al. (13) was used to assess claim accuracy for each post. No language restrictions were applied due to Instagram's built-in translation function. Duplicate posts and those unrelated to toothpaste were excluded.

### Data analysis

To assess data normality, descriptive statistics, visual plots (Q-Q plots and histograms), and normality tests were used. As all data exhibited non-normal distributions, non-parametric analyses were employed. Descriptive statistics included means, standard deviations (SDs), medians, interquartile ranges (IQRs), frequencies, and percentages. Claim accuracy was categorized as factual (objectively true, relevant, and minimal facts) or non-factual (including both non-facts and falsified information). Inferential tests included the Mann-Whitney *U*-test that was used to compare two groups, such as educational vs. promotional content, on metrics like likes, views, and comments. Kruskal-Wallis test was applied for multi-group comparisons, such as examining differences based on poster roles (e.g., patients, dentists, companies). Pairwise comparisons were conducted post-hoc with Bonferroni adjustments to account for multiple comparisons. Chi-square tests was used to analyze categorical data, such as content types (photo vs. video) and themes (marketing vs. educational). Monte Carlo corrections were applied when expected cell counts were less than five to ensure the validity of results. Data was analyzed using IBM SPSS Statistics for Windows, version 22.0 (IBM Corp.). Statistical significance was set at *p* < .05.

## Results

The total number of posts related to the 12 included hashtags was (*n* = 1,054,985), with the highest number under the #Toothpaste hashtag, followed by #Whiteningtoothpaste. In contrast, the lowest number of posts was found on the # ToothpasteNatural hashtag. Table 1 represents the main characteristics of posts under toothpaste-related hashtags. The mean ± SD number of likes or views, comments, and followers were 611.96 ± 1,394.44, 37.36 ± 156.80, and 75,157.04 ± 217,840.78, respectively. Two-thirds of the posts were photos (66.7%), while videos represented one-third of the content (33.3%).

**Table 1:** Characteristics of posts under toothpaste-related hashtags (*n* = 144).

No. of likes/views	Mean ± SD	611.96 ± 1,394.44
	Median (IQR)	57 (536)
No. of comments	Mean ± SD	37.36 ± 156.80
	Median (IQR)	3 (21)
No. of followers	Mean ± SD	75,157.04 ± 217,840.78
	Median (IQR)	7,850 (33,048)
*n* (%)		
Content type	Photo	96 (66.7)
	Video	48 (33.3)
Post role	Patient	56 (38.9)
	Dental interest group	44 (30.6)
	Dentist	15 (10.4)
	Company	29 (20.1)
	Influencer	41 (28.5)
Post content	Educational	56 (38.9)
	Promotional	88 (61.1)
Post theme	Marketing	88 (61.1)
	Sharing experience	31 (21.5)
	Information giving	25 (17.4)
Post type	Practice advertisement	7 (4.9)
	Product advertisement	137 (95.1)
Account type	Clinician	19 (13.2)
	Company	67 (46.5)
	Practice	17 (11.8)
Accuracy of claims	Fact	49 (34.0)
	Non-fact	95 (66.0)

IQR, interquartile range; SD, standard deviation.

Posts were categorized by the poster's role, with patients accounting for 38.9%, dental interest groups composing 30.6%, companies at 20.1%, and dentists at only 10.4%.

Posts content was predominantly promotional (61.1%), whereas educational content constituted 38.9%. While the post theme highlighted marketing as the primary theme (61.1%), followed by sharing experiences (21.5%) and information giving (17.4%).

In post type, most posts fell into the category of product advertisement (95.1%), with practice advertisements accounting for a mere 4.9%. The types of accounts participating in the posting were diversified, with company leading at 46.5%, influencers at 28.5%, clinicians at 13.2%, and practices at 11.8%. As for the accuracy of claims, two-thirds of the posts were based on non-facts (66%), while one-third of the posts were based on facts (34%).

In Table 2, posts with educational content have significantly more likes/views than promotional content, with a significant *p*-value of 0.004. Educational content also has more comments than promotional content, with a significant difference (*p* = 0.014). No significant difference was observed in the number of followers between educational and promotional posts, with a *p*-value of 0.632.

**Table 2:** Comparison of post characteristics according to post content.

		Educational	Promotional	*p*-value
No. of likes/views	Mean ± SD	876.05 ± 1,690.28	443.90 ± 1,147.36	.004*
	Median (IQR)	120 (749)	36 (194)	
No. of comments	Mean ± SD	70.66 ± 244.33	16.17 ± 38.81	.014*
	Median (IQR)	9.5 (46)	2.5 (13)	
No. of followers	Mean ± SD	55,881 ± 100,384.952	87,423.61 ± 266,903.77	.632
	Median (IQR)	6,779 (85,567)	7,912.5 (28,966)	
Content type: *n* (%)	Photo	28 (29.2)	68 (70.8)	.001*
	Video	28 (58.3)	20 (41.7)	
Post role: *n* (%)	Patient	33 (58.9)	23 (41.1)	<.001*
	Dental interest group	10 (22.7)	34 (77.3)	
	Dentist	11 (73.3)	4 (26.7)	
	Company	2 (6.9)	27 (93.1)	
Post theme: *n* (%)	Marketing	0 (0)	88 (100)	<.001*
	Sharing experience	31 (100)	0 (0)	
	Information giving	25 (100)	0 (0)	
Post type: *n* (%)	Practice advertisement	7 (100)	0 (0)	.001*
	Product advertisement	49 (35.8)	88 (64.2)	
Account type: *n* (%)	Clinician	14 (73.7)	5 (26.3)	<.001*
	Company	6 (9)	61 (91)	
	Practice	16 (94.1)	1 (5.9)	
	Influencer	20 (48.8)	21 (51.2)	
Accuracy of claims: *n* (%)	Fact	19 (38.8)	30 (61.2)	.984
	Non-fact	37 (38.9)	58 (61.1)	

IQR, interquartile range; SD, standard deviation.

* Significant at *P* < .05.

There was a significant difference in the distribution of photo and video content types between educational and promotional posts, with photos being more common in promotional content (*p* = 0.001). The role of the post also differed significantly, with patient and dentist posts being more associated with educational content. At the same time, dental interest groups and companies were more concerned with promotional content (*p* < 0.001).

In post themes and types, all marketing-themed posts were promotional (*p* < 0.001), whereas posts all sharing experiences or giving information were categorized as educational. Practice advertisements were only associated with educational content, while product advertisements were more common in promotional content (*p* = 0.001).

In account types, clinician accounts posted more educational content (*p* < 0.001), while company accounts were predominantly associated with promotional content. Practice accounts primarily posted educational content, and influencers were split relatively even between educational and promotional content.

There was no significant difference in the accuracy of claims between educational and promotional content, with similar distributions of fact (educational: 38.8%, promotional: 61.2%) and non-fact claims (educational: 38.9%, promotional: 61.1%) across both types of content (*p* = 0.984).

In Table 3, a comparison of post characteristics according to the role of the poster has been conducted. The mean number of likes/views for patients, dental interest groups, dentists, companies, and *p*-values are reported as 580.16 ± 1,282.14, 901.39 ± 1,840.28, 674.47 ± 1,439, 201.90 ± 394.2, and 0.232, respectively. The median and interquartile ranges (IQR) have also been reported, with patients and dentists showing a median of 60 and 80 with an IQR of 551 and 250, indicating a wide variability in engagement. The mean values for comments follow a similar trend, with patients and dentists having notably lower mean and median (IQR) values [27.07 ± 49.734 vs. 26.16 ± 58.64 and 7(31) vs. 2(20), respectively] compared to the dental interest group and company. The *p*-value for comments was non-significant at 0.822.

**Table 3:** Comparison of post characteristics according to role of poster.

		Patient	Dental interest group	Dentist	Company	*P*
No. of likes/views	Mean ± SD	580.16 ± 1,282.14	901.39 ± 1,840.28	674.47 ± 1,439	201.90 ± 394.2	.232
	Median (IQR)	60 (551)	70.5 (717)	80 (250)	29 (67)	
No. of comments	Mean ± SD	27.07 ± 49.734	71.41 ± 274.69	26.13 ± 58.64	11.38 ± 17.73	.822
	Median (IQR)	7 (31)	3.5 (20)	2 (20)	3 (14)	
No. of followers	Mean ± SD	43,150.86 ± 94,156.09	123,219.05 ± 323,489.52	44,423.07 ± 69,088.29	79,937.31 ± 235,883.90	.097
	Median (IQR)	4,078.5 (20,483)	15,550 (110,698)	9,300 (87,755)	7,849 (30,075)	
Content type: *n* (%)	Photo	35 (36.5)	30 (31.3)	6 (6.3)	25 (26)	.016*
	Video	21 (43.8)	14 (29.2)	9 (18.8)	4 (8.3)	
Post content: *n* (%)	Educational	33 (58.9)	10 (17.9)	11 (19.6)	2 (3.6)	<.001*
	Promotional	23 (26.1)	34 (38.6)	4 (4.5)	27 (30.7)	
Post theme: *n* (%)	Marketing	23 (26.1)	34 (38.6)	4 (4.5)	27 (30.7)	<.001*
	Sharing experience	29 (93.5)	1 (3.2)	0 (0.0)	1 (3.2)	
	Information giving	4 (16)	9 (36)	11 (44)	1 (4)	
Post type: *n* (%)	Practice advertisement	0 (0)	3 (42.9)	4 (57.1)	0 (0)	<.001*
	Product advertisement	56 (40.9)	41 (29.9)	11 (8)	29 (21.2)	
Account type: *n* (%)	Clinician	0 (0)	4 (21.1)	15 (78.9)	0 (0)	<.001*
	Dental company	5 (7.5)	33 (49.3)	0 (0)	29 (43.3)	
	Dental practice	11 (64.7)	6 (35.3)	0 (0)	0 (0)	
	Influencer	40 (97.6)	1 (2.4)	0 (0)	0 (0)	
Accuracy of claims: *n* (%)	Fact	12 (24.5)	19 (38.8)	11 (22.4)	7 (14.3)	.001*
	Non-fact	44 (46.3)	25 (26.3)	4 (4.2)	22 (23.2)	

IQR, interquartile range; SD, standard deviation.

* Significant at *P* < 0.05.

The number of followers shows a considerable range across the groups, with the mean number of followers for the dental interest group reported as 123,219.05 ± 323,489.52 and a higher median and IQR for dentists at 15,550 and 110,698, respectively. The *p*-value was non-significant at 0.097.

Content type analysis revealed that photos and videos are used variably across the groups, with a notable proportion of educational content in the patient group (58.9%) and promotional content in the dental interest group (38.6%). Certain content types were significant, with a *p-*value of less than 0.001.

Post themes also show variation, with a high percentage of sharing experience posts in the patient group (93.5%). Marketing posts were more frequent in dental interest groups (38.6%) and companies (30.7%). Again, the significance for post themes was significant, with a *p*-value of less than 0.001.

Regarding account types, clinicians and companies show a varied distribution, with influencers having a negligible presence. The accuracy of claims within posts was also assessed, revealing significant differences (*p-*value < 0.001) with an even distribution across factual and nonfactual content. However, the dental interest group presented more factual content (38.8%).

## Discussion

The characteristics of posts under toothpaste-related hashtags showed interesting results; noticeable differences exist between Instagram posts regarding credentials, post format, content, and accuracy of claims, emphasizing the diversity of toothpaste-related content on Instagram.

Posts related to toothpaste hashtags were categorized by the role of the poster, where patients accounted for 38.9%, dental interest groups composed 30.6%, dental company at 20.1%, and dentists only at 10.4%. Instagram posts on dental products can vary significantly between dental patients and dentists. Patients often share their experiences with dental treatments, including before-and-after photos, testimonials, and reviews of dental products or procedures. These posts by patients tend to focus on personal experiences, visual outcomes, and the overall impact of dental treatments on their lives. Patients may use hashtags related to their treatment, share their journey to oral health improvement, and seek advice or support from the dental community on Instagram (14). On the other hand, dentists' Instagram posts related to dental products are more likely to be educational, informative, and professional in nature. Dentists may use their posts to showcase their expertise, share insights on dental procedures, provide oral health tips, and promote dental products or services. These posts by dentists often aim to educate their audience, build credibility, and attract potential patients to their practice. Dentists may also use Instagram to demonstrate their work, share case studies, and engage with their followers on oral health topics (14).

This study highlights the influential role of Instagram in shaping consumer perceptions and behaviors related to toothpaste selection. The predominance of promotional content over educational material underscores a significant concern: marketing agendas often overshadow public health education. Despite promotional posts dominating the platform, educational posts consistently garnered higher engagement, including more likes and comments. Promotional posts typically focus on showcasing products, services, or brands with the intent to drive sales or increase brand visibility and awareness (15). This finding suggests that consumers value and seek out reliable, informative content about oral health but are underserved in this regard.

On the other hand, educational posts aim to inform, educate, and raise awareness about oral health, dental procedures, and preventive care. Educational content on Instagram may include tips, facts, tutorials, or informative graphics to empower followers with knowledge and promote oral health literacy (16). Research has shown that promotional posts tend to be more prevalent on Instagram, focusing on marketing products or services (15). These posts may include advertisements, product showcases, or influencer endorsements to generate interest and drive consumer engagement. In contrast, although less common, educational posts are crucial in providing valuable information to followers, fostering trust, and establishing credibility for dental professionals and brands (16).

A critical issue revealed in this study is the limited presence of fact-based posts, with only one-third meeting accuracy standards. This highlights a pervasive issue of misinformation, potentially leading to misguided consumer choices. The high proportion of non-factual claims, especially in promotional posts, raises ethical concerns about compliance with advertising guidelines and underscores the need for regulatory oversight. Such oversight could include stricter content guidelines for influencers and companies, emphasizing transparency and accuracy in claims about dental products. In the realm of dental products, fact-based Instagram posts often feature information substantiated by scientific research, such as the efficacy of specific toothpaste components supported by studies by Murray et al. These posts aim to inform followers with precise and evidence-based details regarding oral health and dental products. Conversely, non-fact-based posts might showcase personal stories, anecdotal evidence, or promotional material lacking scientific validation (17). Through critically evaluating Instagram posts' content and information sources, users can differentiate between fact-based content offering reliable information and non-fact-based content lacking evidence or credibility. This differentiation is vital for making well-informed oral health and dental product decisions based on precise and trustworthy information.

Content type analysis revealed that photos and videos are used variably across the groups where dental companies tend to rely more on photos than videos for promotional purposes on Instagram than dentists and patients. While Instagram allows for photo and video content, studies have shown a preference for photos in certain industries. This finding aligns with a previous study stating that photos attract more likes than other posts (18). For instance, a study on plastic surgeons' engagement on Instagram found that personal and comedic videos garnered higher follower engagement than promotional videos ^(^14, 18). Additionally, research on orthodontics-related posts on Instagram highlighted that dental professionals mainly use the platform rather than for advertising (19). This suggests dental companies may prioritize sharing visual content, such as photos, to showcase their work and engage with their audience on Instagram. Moreover, the type of content posted can influence follower involvement on Instagram. Posts related to events and promotions led to greater follower engagement on the platform (20). This indicates that dental companies may choose to focus on visually appealing posts like photos to attract and engage their audience effectively. While videos can be engaging, especially for certain demographics like young adults (21), the current trend among dental companies on Instagram seems to lean towards utilizing photos for promotional purposes. By leveraging the visual nature of Instagram and focusing on high-quality photos, dental companies can effectively showcase their services and engage with their audience on the platform.

A concern to be raised is how this influence affects the consumers and whether this effect is positive or negative regarding improving oral health (22); it was noticed that most posts about toothpaste were concerning their safety, particularly the fluoridated toothpaste with an emphasis on fluoride arm effect, and the need to use herbal or even homemade toothpaste. Regarding public opinion, fluoride toothpaste prevents dental caries, especially in children and adolescents. Fluoride toothpaste has been extensively studied and proven effective in preventing dental caries (23). The efficacy of fluoride toothpaste in caries prevention has been well-established, with significant benefits observed when using fluoride concentrations of 1,000 ppm and above (24). The FDA and ADA recommendations emphasize the importance of fluoridated toothpaste with appropriate fluoride concentrations for effective caries prevention. The ADA recommends using conventional fluoride toothpaste with concentrations ranging from 1,000 to 1,500 ppm fluoride for individuals of all ages, except for infants (25). These guidelines aim to ensure fluoridated toothpaste's safe and beneficial use across different age groups, emphasizing the significance of oral health practices supported by scientific evidence and regulatory oversight. From the results of this study, only 10% of the highly liked/viewed posts were by dentists, and only 34% were fact posts; meanwhile, 61.1% were promotional. Given that the influencers or promoting agency is driven by marketing and increasing sales agendas; concerns about compliance with advertising regulations had to be raised. Fluoridated Toothpaste holds a market share of over 95% among over-the-counter toothpaste products (26). Such high stakes may impact the influencer's posts, causing them not to always adhere to guidelines (27).

Various promotion strategies, including influencer marketing, visual storytelling, and engagement tactics, influence Instagram posts related to dental products. Influencer marketing on Instagram has positively impacted consumer behavior toward the marketed brand, leading to increased product sales and brand awareness (28, 29). Collaborating with influencers who resonate with the target audience can effectively promote dental products like toothpaste, leveraging the influencers' credibility and reach to enhance brand visibility and engagement.

Visual storytelling plays a crucial role in promoting dental products on Instagram. Through visually appealing posts, informative captions, and interactive stories, dental brands can effectively communicate the benefits of their products and engage with their audience (30). This approach helps increase awareness about oral hygiene practices and educates followers about the importance of oral health.

Instagram followers' trust in influencers is often linked to the perceived accuracy of the information they provide, influencing decisions such as product choice and oral health behavior (31). Moreover, engagement with influencer content on Instagram can be substantial, with influencers strategically managing their posts to attract viewership and engagement (32). This strategic curation of content raises questions about the authenticity and accuracy of the information shared. There is a need to raise public awareness of the necessity to obtain a balance between posts by educators, dentists, and influencers/promoters.

Practical implications extend beyond the dental profession. Regulators and social media platforms should implement measures to verify and prioritize fact-based health-related content. Collaborations between dental associations and influencers could help leverage their wide-reaching platforms to disseminate accurate, educational messages. Additionally, educational campaigns tailored for social media could capitalize on Instagram's visual appeal and engagement-driven algorithms to promote public oral health literacy. From a marketing perspective, companies should recognize the potential value of integrating educational elements into their promotional strategies. Posts combining engaging visuals with credible, educational content could build consumer trust while meeting marketing objectives.

Recommendations for improving the impact of Instagram in promoting oral health involve several key stakeholders. Dental professionals should enhance their presence on social media by creating content that simplifies oral health education while ensuring scientific accuracy. Regulators need to establish and enforce clear advertising guidelines to guarantee the accuracy of promotional posts, particularly those shared by influencers. Social media platforms should introduce fact-checking mechanisms for health-related posts and refine their algorithms to prioritize educational content over purely promotional material. Companies, in turn, can integrate evidence-based educational elements into their marketing strategies to build trust and credibility with consumers.

## Limitations

This study has several limitations that may influence the generalizability and interpretation of its findings. One key limitation is the reliance on Instagram's algorithm to select the “Top 12 posts” for each hashtag. This method, while practical for identifying high-impact content, inherently introduces selection bias, as it favors posts that have already gained visibility and engagement due to Instagram's amplification mechanisms. As a result, less popular but potentially informative or accurate posts may have been overlooked. In addition, the data collection was conducted on a single day, which presents temporal bias. Social media content is highly dynamic, with trends, seasonality, and current events potentially influencing the nature of posts. For instance, campaigns or promotional efforts by companies may temporarily dominate content under specific hashtags, skewing the data toward certain themes or types of posts. Another limitation lies in the exclusion of posts not tagged with the selected hashtags. While hashtags are a useful means of categorizing content, many relevant posts may not include these specific hashtags, leading to incomplete data representation. Furthermore, linguistic and cultural diversity among Instagram users presents a challenge, as some content may not have been adequately interpreted or evaluated due to language barriers or differences in context. The study also acknowledges potential biases introduced by Instagram's recommendation algorithms. Even though a newly created account was used to minimize personalization bias, the algorithm's inherent preferences for certain types of content (e.g., visually engaging posts or those by popular influencers) may still have influenced the results. Last but not least, the study's assessment of claim accuracy relied on a simplified 2-point scale, which, while practical, may not fully capture the nuances of content quality or the subtleties of misinformation. Posts categorized as “non-factual” may still contain some elements of truth, and those marked as “factual” might lack sufficient context or explanation to support comprehensive understanding.

Future research should consider longitudinal data collection to better account for temporal variations and include a broader range of hashtags and untagged content. Moreover, the development of more detailed and robust criteria for evaluating claim accuracy could enhance the precision and reliability of such analyses. By addressing these limitations, future studies can provide a more comprehensive understanding of social media's role especially Instagram in shaping consumer behavior and public health education.

## Conclusion

Instagram shapes consumer behavior in toothpaste selection, with promotional content dominating despite higher engagement with educational posts. Although Instagram influencers play a significant role in shaping consumer behavior and perceptions, concerns regarding the content's accuracy, quality, and compliance continue to rise. Limited fact-based content highlights the need for stricter regulations and increased contributions from dental professionals to improve oral health education. As social media continues to influence various aspects of society, further research is necessary to ensure the reliability and integrity of information disseminated through these platforms.

## Data Availability

The raw data supporting the conclusions of this article will be made available by the authors, without undue reservation.
